# From theory to practice: using the Normalization Process Theory and Theoretical Domains Framework to understand implementation of decarbonization in general practice

**DOI:** 10.1093/fampra/cmaf050

**Published:** 2025-07-02

**Authors:** Ana Raquel Nunes, Helen Atherton, Frederik Dahlmann, Abi Eccles, Olivia Geddes, Michael Gregg, Florence Karaba, Rachel Spencer, Helen Twohig, Jeremy Dale

**Affiliations:** Warwick Medical School, Medical School Building, University of Warwick, Coventry, CV4 7AL, United Kingdom; School of Primary Care, University of Southampton, University Road, Southampton, SO16 5ST, United Kingdom; Warwick Business School, University of Warwick, Scarman Road, Coventry, CV4 7AL, United Kingdom; Nuffield Department of Primary Care Health Sciences, University of Oxford, Radcliffe Primary Care Building, Radcliffe Observatory Quarter, Woodstock Road, Oxford. OX2 6GG, United Kingdom; Warwick Medical School, Medical School Building, University of Warwick, Coventry, CV4 7AL, United Kingdom; PPI co-investigator, GPNET-0 Study, United Kingdom; Warwick Medical School, Medical School Building, University of Warwick, Coventry, CV4 7AL, United Kingdom; Warwick Medical School, Medical School Building, University of Warwick, Coventry, CV4 7AL, United Kingdom; School of Medicine, David Weatherall building University Road, Keele University, Staffordshire, ST5 5BG, United Kingdom; Warwick Medical School, Medical School Building, University of Warwick, Coventry, CV4 7AL, United Kingdom

**Keywords:** decarbonization, sustainability, net zero healthcare, behaviour change, emissions reduction, climate action, environmental sustainability, planetary health

## Abstract

**Background:**

Decarbonization in general practice is a critical step toward achieving a net zero healthcare system. Understanding the factors that facilitate or hinder the implementation of environmentally sustainable practices is essential for effective and equitable action. Hence, the overarching aim of this study is to advance understanding of the factors influencing decarbonization in general practice. This study’s objective is to map and compare the application of the Normalization Process Theory (NPT) and Theoretical Domains Framework (TDF) in understanding the key factors and sub-factors related to decarbonization in general practice.

**Methods:**

Factors derived from a systematic review and narrative synthesis were mapped to NPT constructs and TDF domains by a multidisciplinary team of 10 coders, including academic general practitioners (GPs), researchers, and patient representatives. The mapping was conducted independently, and coder agreement was evaluated for consistency and reliability in categorization.

**Results:**

The study identifies key NPT (‘Coherence’, ‘Collective Action’, and ‘Cognitive Participation’) and TDF domains (‘Environmental Context and Resources’, ‘Knowledge’, and ‘Social/professional role and identity’) associated with factors identified in previous research as being associated with achieving decarbonization in general practice. A high intercoder reliability rate (73% for NPT, 84% for TDF) supports the consistency of the analysis, particularly for structured drivers such as financial incentives and policy support.

**Conclusions:**

The findings demonstrate that the NPT and TDF frameworks provide useful, though incomplete, insights into factors influencing decarbonization in general practice. Such factors require more attention when developing evidence-based strategies for promoting decarbonization, something that future research could evaluate.

**Key messages**

- The Normalization Process Theory (NPT) and Theoretical Domains Framework (TDF) are effective in capturing key factors influencing decarbonization in general practice.
- High intercoder reliability confirms the consistency and suitability of using the NPT and TDF to categorize factors related to decarbonization in general practice.
- Variability in interpreting subjective factors shows the need for refinement of existing frameworks.
- Current literature gaps reveal a lack of research on emotional and motivational aspects of decarbonization in general practice.
- Using a multidisciplinary approach strengthens the reliability of study’s findings.

## Background

Climate change poses a profound threat to global health with far-reaching implications for disease patterns, health systems, and societal well-being [1–6]. Recognizing this, healthcare systems worldwide are under increasing pressure to reduce their environmental impact [7–16]. In the UK, the National Health Service (NHS) has committed to achieving net zero emissions by 2040 [17], placing general practice, a cornerstone of primary healthcare, at the forefront of decarbonization efforts [18–21]. Given its scale and accessibility, general practice has the potential to drive meaningful environmental change while delivering critical health services [22, 23].

In the context of this study, decarbonization refers to initiatives to improve the environmental sustainability of general practice; these include measures implemented to reduce greenhouse gas emissions, targeting clinical, operational, and organizational activities [17]. Understanding the factors that influence the implementation of decarbonization actions within general practice is crucial for developing effective strategies to mitigate climate change and its health consequences [24]. There are likely to be multifaceted challenges, including structural, organizational, and individual-level barriers, as well as patient-related dynamics [25–27]. Environmental goals need to be balanced with clinical outcomes, patient care quality, and operational feasibility [28–31].

A recent systematic review undertaken by our team highlighted the multifaceted nature of decarbonization, spanning micro, meso, and macro system levels, each with its clinical practices or procurement processes [32, 33]. The review examined 15 studies spanning five countries, with the majority originating from the UK (*n* = 5), followed by Australia (*n* = 3), the USA (*n* = 2), Germany (*n* = 2), and one each from France, Switzerland, and Israel. These studies used various methodologies (qualitative, *n* = 7; quantitative, *n* = 7; mixed methods, *n* = 1), and included a range of participants, such as healthcare professionals (*n* = 7), patients (*n* = 5), health stakeholders (*n* = 2), and the general public (*n* = 1). Four key factors were identified: (i) institutional and policy support; (ii) organizational leadership, support, and constraints; (iii) professional knowledge, awareness, and engagement; and (iv) patient and community engagement. Within these, 15 sub-factors provided nuanced insights into enablers and barriers to sustainable practices [32]. Strategies to tackle decarbonization in general practice need to navigate entrenched systems, overcome organizational and individual inertia, and balance varying stakeholder priorities [33].

This study builds on these findings to further enhance understanding of decarbonization in general practice. Using a mapping exercise, we sought to identify how factors from our systematic review aligned with NPT and TDF constructs to gain deeper insights into the mechanisms enabling or hindering such actions. Frameworks such as the Normalization Process Theory (NPT) [34–36] and Theoretical Domains Framework (TDF) [37] offer structured approaches to understanding behaviour change at organizational and individual levels. The NPT focuses on the social processes and interactions that facilitate or hinder the normalization of new practices within systems [34–36]. On the other hand, the TDF identifies cognitive, affective, and environmental determinants of behaviour, providing a comprehensive lens for analysing professional knowledge, individual engagement, and contextual factors [37]. Together, these frameworks enable a holistic analysis of individual and systemic factors, capturing the complexity of embedding the implementation of new practices in healthcare. These frameworks have been used effectively in previous studies to explore the implementation processes in primary care [38] and design interventions for behaviour change in healthcare [39], including interventions targeting clinical practice, patient engagement, and system-level transformations [40].

Hence, the overarching aim of this study is to advance understanding of the factors influencing decarbonization in general practice. Specifically, this study’s objective is to map and compare the application of the NPT and TDF in understanding key factors and sub-factors related to decarbonization.

## Methods

We conducted a multistep mapping exercise that drew on the interpretations of a multidisciplinary team of coders to align identified factors from the systematic review with the relevant constructs of NPT [34–36] and domains of TDF (version 2) [37]. Coders (see coders characteristics in Section 3.1) independently coded a dataset consisting of four main factors and fifteen sub-factors (Table 1) derived from a recent systematic review [33], mapping them onto NPT core constructs and TDF domains (Supplementary Table S1). This process aimed to ensure diverse perspectives in assigning alignment.

**Table 1.** Decarbonization factors derived from the literature [33] mapped to the NPT and TDF.

Factors [33]	NPT core construct (% agreement)^*^	TDF domain (% agreement)^*^
1. Institutional and policy support		
1.1. Financial incentives and policies Financial incentives are essential for the adoption of decarbonization actions, but inconsistent policy guidance in some regions acts as a barrier.	[3] Collective Action (80%)	[7] Reinforcement (50%); [11] Environmental context and resources (100%)
1.2. Frameworks and declarations Guidelines such as the WONCA declaration motivate GPs to integrate climate change considerations into their practices by providing structured guidelines and strategic vision.	[1] Coherence (60%) [3] Collective Action (40%)	[1] Knowledge (50%); [3] Social/professional role and identity (40%); [11] Environmental context and resources (70%)
1.3. System-level changes Effective decarbonization requires better networking and centralization of sustainability efforts to ensure coherence and efficiency across the healthcare system.	[1] Coherence (50%); [3] Collective Action (70%)	[11] Environmental context and resources (80%) [12] Social influences (60%)
2. Organizational leadership, support, and constraints		
2.1. Leadership and culture Proactive leadership and a culture that values sustainability are critical for driving successful decarbonization efforts within general practices.	[1] Coherence (40%); [2] Cognitive Participation (70%)	[3] Social/professional role and identity (60%); [11] Environmental context and resources (80%); [12] Social influences (40%)
2.2. Practice management Effective leadership and staff engagement are essential for integrating decarbonization actions into daily practice activities.	[2] Cognitive Participation (80%)	[3] Social/professional role and identity (50%); [11] Environmental context and resources (60%)
2.3. Resource constraints High costs and resource limitations hinder the adoption of sustainable measures, requiring financial support and cost-effective solutions.	[3] Collective Action (70%)	[11] Environmental context and resources (100%)
3. Professional knowledge, awareness, and engagement		
3.1. Knowledge and awareness Clinician awareness of climate change impacts is crucial, but many lack specific knowledge and feel uncomfortable discussing it with patients.	[1] Coherence (90%)	[1] Knowledge (100%); [4] Beliefs about capabilities (50%)
3.2. Education and training Enhancing clinician competence through targeted education and training on decarbonization is needed.	[1] Coherence (80%)	[2] Skills (90%); [6] Beliefs about consequences (40%)
3.3. Personal environmental consciousness GPs who are environmentally conscious personally are more likely to adopt decarbonization actions professionally.	[1] Coherence (60%); [2] Cognitive Participation (60%)	[1] Knowledge (40%); [3] Social/professional role and identity (60%); [4] Beliefs about capabilities (50%); [6] Beliefs about consequences (40%)
3.4. Variation in awareness and engagement Significant differences exist among clinicians, with a high willingness to learn but low comfort in counselling patients on climate-related issues.	[1] Coherence (50%) [2] Cognitive Participation (40%)	[3] Social/professional role and identity (60%) [6] Beliefs about consequences (50%)
3.5. Preferences and acceptance Variability in acceptance of sustainability roles and measures, with constraints including limited awareness, funding, and patient motivation.	[1] Coherence (40%); [2] Cognitive Participation (70%)	[1] Knowledge (50%); [3] Social/professional role and identity (50%); [11] Environmental context and resources (50%)
4. Patient and community engagement		
4.1. Patient discussions and barriers Many GPs discuss climate change with patients, but barriers such as time constraints and lack of recommendations limit these discussions.	[3] Collective Action (70%)	[4] Beliefs about capabilities (40%); [11] Environmental context and resources (90%)
4.2. Patient perception and information sources Patients believe climate change affects health but rely on non-medical sources for information.	[1] Coherence (90%)	[1] Knowledge (60%); [12] Social influences (50%)
4.3. Community engagement in activities Local communities engage in nature-based activities, but awareness of initiatives like Green Social Prescribing is limited.	[1] Coherence (50%)	[1] Knowledge (80%)
4.4. Information gap Patients trust physicians but do not view them as primary sources of environmental information, relying instead on news outlets, social media, and personal networks.	[1] Coherence (40%)	[1] Knowledge (60%); [3] Social/professional role and identity (50%); [12] Social influences (40%)

^*^The domains presented were the ones that most coders aligned with NPT or TDF. Agreement was evaluated using IRR values, with IRR ≥ 81% interpreted as almost perfect agreement, values between 61% and 80% as substantial agreement, and values between 40% and 60% as moderate. Scores below 40% were considered fair or poor agreement and have not been included.

Intercoder reliability was measured to evaluate agreement levels among coders in the mapping process, with agreement rates calculated using established guidelines [41].

### Procedure

The coders were briefed on the study’s aims and the structured insights that the frameworks provide, framing the exercise as an exploration of mechanisms driving or hindering decarbonization. Each coder then received a mapping table detailing the main and sub-factors influencing decarbonization (Supplementary Table S1), along with reference materials outlining NPT core constructs (Supplementary Table S2) and TDF domains (Supplementary Table S3). All coders were familiar with the NPT and TDF as part of their involvement in a longitudinal study [24] where these frameworks are integral to data collection and analysis. They were asked to independently align the factors with the NPT constructs and TDF domains, documenting their choices. Coders could map the data to multiple domains when needed. This allowed a richer and more accurate representation of the data. A constructivist perspective was adopted [42], encouraging coders to actively interpret the factors based on the frameworks.

### Data analysis

The analysis followed a deductive approach using NPT and TDF to align with the study’s focus on implementation and behavioural factors. After coding, the degree of agreement among coders was calculated using an inter-rater reliability (IRR) metric. This was expressed as a percentage, representing the proportion of coders who independently mapped a given factor to the same NPT construct or TDF domain. To calculate the IRR, the total number of agreements between coders for each mapping was divided by the total number of possible coder comparisons for that factor, providing a measure of consensus [41]. The aggregated results were analysed to identify consistent alignments, highlighting the factors most robustly linked to specific constructs in the NPT and TDF frameworks. This approach ensured transparency and reliability in determining the strength of associations between factors and theoretical constructs.

## Results

### Coder characteristics

The study team included ten coders: three academic general practitioners (GPs) (two clinically active), six multidisciplinary researchers working in universities, and one patient representative. Supplementary Table S4 provides details on their background, including research and clinical experience.

### Intercoder reliability

Intercoder reliability was assessed to ensure consistency in mapping factors to the frameworks. NPT constructs yielded an intercoder reliability rate (IRR) of 73%, while TDF domains achieved 84%. Consistent with established benchmarks, IRR ≥ 81% was interpreted as almost perfect agreement, values between 61% and 80% as substantial agreement, and values between 40% and 60% as moderate [41]. Scores below 40%, were considered fair or poor agreement and have not been included [41]. Table 1 reports the factors from the review that mapped against the constructs and domains with the highest coder agreement.

### Institutional and policy support

**Figure 1.**
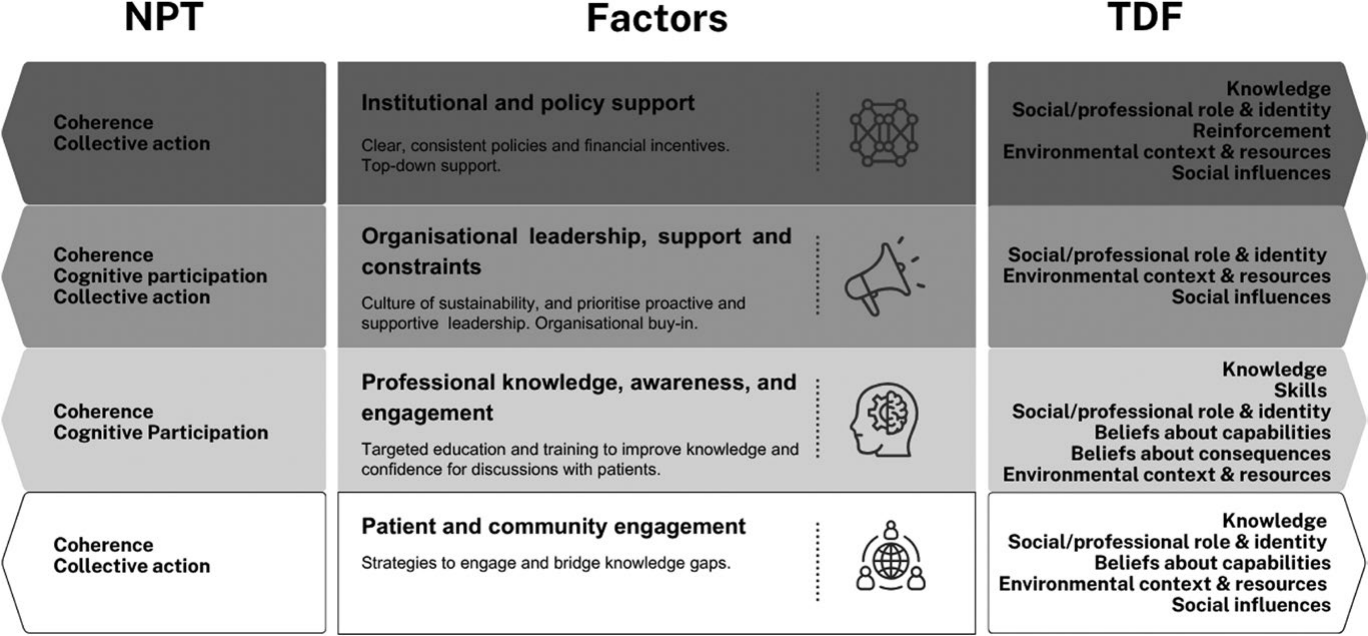
Summary of mapping.

There was strong agreement that financial incentives and policies align with “Collective action” in NPT (80%) (see Supplementary Table S1 for description of NPT constructs) and “Environmental context and resources” (100%) in TDF, while a moderate agreement was found for their alignment with “Reinforcement” (50%) in TDF (see Supplementary Table S3 for description of TDF domains) (Table 1, Fig. 1).

Frameworks and declarations such as the World Organization of Family Doctors (WONCA) guidelines, were linked to ‘Coherence’ (60%) and ‘Collective action’ (40%) in NPT; and to ‘Environmental context and resources’ (70%), ‘Knowledge’ (50%) and ‘Social/professional role and identity’ (40%) in TDF. System-level changes were associated with ‘Coherence’ (50%) and ‘Collective action’ (70%) in NPT, as well as ‘Environmental context and resources’ (80%) and ‘Social influences’ (60%) in TDF. However, no evidence was mapped against ‘Reflexive monitoring’ in NPT.

### Organizational leadership, support, and constraints

There was agreement that leadership and culture align with ‘Cognitive participation’ (70%) and ‘Coherence’ (40%) in NPT, and with ‘Environmental context and resources’ (80%), ‘Social/professional role and identity’ (60%) and ‘Social influences’ (40%) in TDF (Table 1, Fig. 1). Practice management, encompassing team coordination and logistical support, was also linked to ‘Cognitive participation’ (80%) in NPT, and to ‘Environmental context and resources’ (60%) and ‘Social/professional role and identity’ (50%) in TDF. Resource constraints were strongly mapped to ‘Collective action’ (70%) in NPT, and to ‘Environmental context and resources’ (100%) in TDF. However, domains such as ‘Behaviour regulation’ and ‘Beliefs about consequences’ in TDF were absent in this category.

### Professional knowledge, awareness, and engagement

Coders strongly agreed that knowledge and awareness align with ‘Coherence’ (90%) in NPT, and with ‘Knowledge’ (100%) and ‘Beliefs about capabilities’ (50%) in TDF (Table 1, Fig. 1). Education and training were associated with ‘Coherence’ (80%) in NPT, and with ‘Skills’ (90%) and ‘Beliefs about capabilities’ (40%) in TDF. Personal environmental consciousness was linked to ‘Coherence’ (60%) and ‘Cognitive participation’ in NPT (60%), and to ‘Social/Professional role and identity’ (60%), ‘Beliefs about capabilities’ (50%), and ‘Beliefs about consequences’ (40%) in TDF. Variation in awareness and engagement was mapped to ‘Coherence’ (50%) and ‘Cognitive participation’ (40%) in NPT, as well as ‘Social/professional role and identity’ (60%) and ‘Beliefs about capabilities’ in TDF (50%). Preferences and acceptance were linked to ‘Cognitive participation’ (70%) and ‘Coherence’ (40%) in NPT, and to ‘Knowledge’ (50%), ‘Social/professional role and identity’ (50%), and ‘Environmental context and resources’ (50%) in TDF. However, there was limited alignment with domains such as ‘Emotion’, ‘Intentions’, and ‘Behaviour regulation’.

### Patient and community engagement

There was agreement that patient discussions and barriers align with ‘Collective action’ in NPT (70%), and with ‘Environmental context and resources’ (90%) and ‘Beliefs about capabilities’ (40%) in TDF (Table 1, Fig. 1). Patient perception and information sources were mapped to ‘Coherence’ (90%) in NPT, and to ‘Knowledge’ (60%) and ‘Social influences’ (50%) in TDF. Community engagement in activities was linked to ‘Coherence’ in NPT (50%) and to ‘Knowledge’ (80%) in TDF. The information gap, reflecting patients’ reliance on non-medical sources for environmental information, was aligned with ‘Coherence’ (40%) in NPT and to ‘Knowledge’ (60%), ‘Social/professional role and identity’ (50%), and ‘Social influences’ (40%) in TDF. There was no evidence from the review that mapped to constructs such as ‘Reflexive monitoring’ in NPT and domains such as ‘Intentions’ and ‘Behaviour regulation’ in TDF.

### NPT core constructs

The most frequently aligned NPT constructs were ‘Coherence’ (11 occurrences, 40%–90%), ‘Collective Action’ (five occurrences, 40%–80%), and ‘Cognitive Participation’ (five occurrences, 40%–80%) (Table 1, Fig. 1). ‘Reflexive monitoring’ was absent.

### TDF domains

The most frequently aligned TDF domains were ‘Environmental Context and Resources’ (eight occurrences, 50%–100%), ‘Knowledge’ (seven occurrences, 40%–100%), and ‘Social/professional role and identity’ (seven occurrences, 40%–60%). Other domains with notable alignment included ‘Social influences’ (four occurrences, 40%–60%), ‘Beliefs about capabilities’ (three occurrences, 40%–50%), ‘Beliefs about consequences’ (three occurrences, 40%–60%), and ‘Skills’ (one occurrence, 90%). ‘Reinforcement’ (one occurrence, 50%) appeared less frequently. Domains such as ‘Optimism’, ‘Intentions’, ‘Goals’, ‘Memory, attention and decision processes’, ‘Emotion’, and ‘Behaviour regulation’ were notably absent.

## Discussion

### Summary of findings

The findings suggest that the NPT and TDF frameworks are broadly applicable for understanding factors influencing decarbonization in general practice. The coders’ interpretations of these frameworks aligned well with the key factors identified in the literature review, suggesting that the respective elements of NPT and TDF are relevant for this context. This reinforces the academic and practical value of using these frameworks to structure analysis of decarbonization efforts rather than rejecting them as incomplete or unsuitable. IRR = 73% for NPT and IRR = 84% for TDF indicate substantial agreement, supporting the consistency of the mapping exercise and demonstrating the frameworks’ robustness in capturing the systemic, organizational, and individual-level factors driving decarbonization.

Coders achieved strong agreement in their interpretation of systemic and external drivers such as financial incentives and policy support, which aligns with the NPT construct of ‘Collective action’ and the TDF domain of ‘Environmental context and resources’. Similarly, frameworks and declarations such as the WONCA guidelines were mapped to ‘Coherence’ and ‘Collective action’ in NPT, and to ‘Environmental context and resources’ and ‘Knowledge’ in TDF, further demonstrating consistency in how coders applied these constructs to practical examples.

However, greater variability emerged for factors with more subjective or contextual dimensions, such as personal environmental consciousness, which was mapped to ‘Social/professional role and identity’ in TDF. Similarly, individual and community engagement factors, such as variation in clinicians’ awareness or patients’ reliance on non-medical information sources, showed inconsistent mappings to constructs such as ‘Coherence’ and ‘Cognitive participation’ in NPT and related TDF domains. This variability highlights the challenges of uniformly interpreting subjective elements and highlights areas where further refinement or clarification of the frameworks within the context of decarbonization actions may be needed to ensure consistency.

Moreover, there was high agreement that some constructs and domains did not map against any evidence, pointing to gaps in the literature. For example, ‘Reflexive monitoring’ in NPT, along with ‘Emotion’, ‘Behaviour regulation’, and ‘Intentions’ in TDF, were not associated with any factors from the review. This suggests an under representation of the relevance of iterative evaluation process, emotional and motivational drivers, and behavioural mechanisms in the evidence space. Addressing these gaps through future research could reveal overlooked barriers or facilitators, such as how trust, reflection, and emotional engagement influence the sustained adoption of decarbonization practices in healthcare. Identifying why these constructs and domains remain unaddressed may also help to refine both theoretical frameworks and practical interventions, ensuring a more comprehensive approach to fostering sustainable healthcare practices.

### Strengths

This study applies the NPT and TDF frameworks to the available evidence on decarbonization in general practice, providing a structured approach and a behaviour change lens to understanding the factors influencing adoption and sustainability. The inclusion of a multidisciplinary team of academic GPs, researchers, and patient representatives strengthened the analysis, ensuring the inclusion of diverse perspectives. Allowing coders to map data onto multiple domains of the NPT and TDF enabled a nuanced analysis, capturing the complexity and interrelatedness of concepts across domains. The systematic, protocol-driven methodology improved reliability and uncovered patterns in coder agreement, offering a foundation for future research and interventions.

### Limitations

Some degree of variation and subjectivity is inherent in this type of interpretive exercise. The study’s reliance on evidence from a single systematic review as the data source may also limit the diversity of identified factors. Furthermore, the limited breadth, depth, and quality of the included literature constrains the comprehensiveness of the findings, potentially omitting key insights or nuances relevant to the topic. Despite few coders having formal prior experience applying the NPT and TDF, all coders were familiar with them through their involvement in a longitudinal study [24] where these frameworks are integral to data collection and analysis. A limitation of allowing coders to map data onto multiple domains of the NPT and TDF is the potential loss of specificity. As the analysis followed a deductive approach using NPT and TDF, this may have constrained the identification of novel or context-specific insights beyond the frameworks. The coders found the mapping challenging but valuable, as it improved theoretical grounding and allowed a better understanding of the data.

### Implications for research

To ensure methodological rigour, research should use collaborative approaches with multiple researchers engaging in iterative discussions. This layered scrutiny is essential to address the interpretative complexity of decarbonization strategies.

Future research should prioritize the collection of empirical data from GPs, patients, and other key stakeholders to address gaps identified in this mapping exercise and to confirm emerging patterns [43, 44]. These efforts are critical to building a robust evidence base for driving decarbonization in primary care [45, 46]. The variability in coder agreement on individual-level factors highlights the need to better understand the interplay between professional identity, personal values, and social influences in promoting engagement with decarbonization actions [47–52]. Future research should explore these dynamics to design effective interventions capable of fostering systemic change [25, 53, 54]. Additionally, research should examine how policy designs and funding mechanisms shape the adoption and implementation of decarbonization strategies [43].

## Conclusions

Adopting a systems perspective is essential to comprehensively address the interconnectedness of policies, organizational resources, and individual behaviours. By applying the NPT and TDF frameworks to factors identified from the literature as influencing decarbonization in primary care, this study elucidates pathways to achieving the behaviour change necessary for the sector to reach net zero emissions. The findings highlight the critical roles of institutional support, professional engagement, and community involvement in driving sustainable practices, and gaps in the evidence base that need further research.

## Supplementary Material

cmaf050_suppl_Supplementary_Tables_1-4

## Data Availability

Data will be shared on reasonable request to the corresponding author.

## Patient and public involvement

Our study was developed with extensive patient and public involvement and has two lay representative co-investigators, and a patient and public panel made up of nine individuals who are providing advice and feedback on all aspects of the study. One of the co-authors is a lay representative.
