# Supplementary material for: From theory to practice: using the Normalization Process Theory and Theoretical Domains Framework to understand implementation of decarbonization in general practice
Source: Fam Pract. 2025 Jul 2;42(4):cmaf050. doi: 10.1093/fampra/cmaf050 (PMC12214461; doi:10.1093/fampra/cmaf050)
Supplement: cmaf050_suppl_Supplementary_Tables_1-4 [file cmaf050_suppl_supplementary_tables_1-4.pdf]

Supplementary Materials

Supplementary File 1 - PROTOCOL: Applying the Normalisation Process Theory (NPT) and Theoretical Domains Framework (TDF) to guide the implementation of decarbonisation actions in general practice

Rationale: A systematic review has been conducted aimed at summarising existing literature on the implementation of decarbonisation actions in general practice, to outline the actions being implemented, factors influencing decarbonisation, identify evidence gaps and questions for future research.

The findings from the review have significant implications for health policy, clinical practice, and patient care, aligning well with behaviour change frameworks such as the Theoretical Domains Framework (TDF) and Normalisation Process Theory (NPT). Decarbonisation actions in general practice are influenced by institutional, organisational, and individual behavioural factors, as well as contextual factors like patient views and experiences. Such combined approach will systematically identify cognitive, affective, and environmental determinants relevant to implementing decarbonising actions within general practice and understand the dynamic social processes involved.

To achieve this, the aim is to align these findings with the NPT and TDF frameworks. Applying these frameworks should offer a structured analysis to gain a deeper understanding of underlying mechanisms, and help inform targeted interventions, fostering effective implementation and sustainability of decarbonisation actions in general practice. Using a multi-disciplinary and patient and public involvement (PPI) perspectives within the team is critical to achieve this due to the complex nature of the task and the diverse range of factors influencing decarbonisation efforts in general practice. A constructivist perspective will be adopted, considering each member of the team as actively engaged in constructing meaning based on the identified factors.

Please note that detailed definitions and descriptions of terminologies related to the NPT and TDF frameworks are provided.

Aim: Map the NPT and TDF frameworks onto the implementation factors identified in Table 1.

Methods: You will independently perform the mapping. It is anticipated that this will take about 30 minutes to complete.

Materials:

- Table - Factors influencing the adoption, implementation and integration of decarbonisation actions, and NPT core constructs and TDF domains columns for you to fill in.
- Table – Description of NPT core constructs.
- Table – Description of TDF domains.

Supplementary Table 1. Factors influencing the adoption, implementation and integration of decarbonisation actions.

Factors	Description	NPT core constructs	TDF domains
1. Institutional and policy support			
1.1. Financial incentives and policies	Financial incentives are essential for the adoption of decarbonisation actions, but inconsistent policy guidance in some regions acts as a barrier [35,36].		
1.2. Frameworks and declarations	Guidelines such as the WONCA declaration motivate GPs to integrate climate change considerations into their practices by providing structured guidelines and strategic vision [35,36].		
1.3. System-level changes	Effective decarbonisation requires better networking and centralisation of sustainability efforts to ensure coherence and efficiency across the healthcare system [31,35].		
2. Organisational leadership, support, and constraints			
2.1. Leadership and culture	Proactive leadership and a culture that values sustainability are critical for driving successful decarbonisation efforts within general practices [31,41].		
2.2. Practice management	Effective leadership and staff engagement are essential for integrating decarbonisation actions into daily practice activities [31,41].		
2.3. Resource constraints	High costs and resource limitations hinder the adoption of sustainable measures, requiring financial support and cost-effective solutions [31,35].		
3. Professional knowledge, awareness, and engagement			
3.1. Knowledge and awareness	Clinician awareness of climate change impacts is crucial, but many lack specific knowledge and feel uncomfortable discussing it with patients [32,37,38].		
3.2. Education and training	Enhancing clinician competence through targeted education and training on decarbonisation is needed [32,43].		
3.3. Personal environmental consciousness	GPs who are environmentally conscious personally are more likely to adopt decarbonisation actions professionally [32,35].		

3.4. Variation in awareness and engagement	Significant differences exist among clinicians, with high willingness to learn but low comfort in counselling patients on climate-related issues [37,38].		
3.5. Preferences and acceptance	Variability in acceptance of sustainability roles and measures, with constraints including limited awareness, funding, and patient motivation [41,42].		
4. Patient and community engagement			
4.1. Patient discussions and barriers	Many GPs discuss climate change with patients, but barriers such as time constraints and lack of recommendations limit these discussions [34,37,38].		
4.2. Patient perception and information sources	Patients believe climate change affects health but rely on non-medical sources for information [37,38].		
4.3. Community engagement in activities	Local communities engage in nature-based activities, but awareness of initiatives like Green Social Prescribing is limited [42,44].		
4.4. Information gap	Patients trust physicians but do not view them as primary sources of environmental information, relying instead on news outlets, social media, and personal networks [37,38].		

Supplementary Table 2: NPT Core Constructs

Normalization Process Theory | NPT Core Constructs (northumbria.ac.uk) ; Normalization Process Theory | NPT Toolkit (northumbria.ac.uk)

NPT Core Constructs	
**1. Coherence** is the sense-making work that people do individually and collectively when they are faced with the problem of operationalizing some set of practices. Like all NPT constructs it has four components.	1.1 Differentiation: An important element of sense-making work is to understand how a set of practices and their objects are different from each other. *For example, when doctors use a videoconferencing system to consult with patients, what do they do to understand and organize the differences between face-to-face consultations and videoconferencing.*
	1.2 Communal specification: Sense-making relies on people working together to build a shared understanding of the aims, objectives, and expected benefits of a set of practices. *A great example is the team of investigators leading a clinical trial, as they work out how to integrate a complex clinical experiment into a healthcare setting, and as they try to identify and anticipate the relationship between elements of the trial and everyday clinical practice.*
	1.3 Individual specification: Sense-making has an individual component too. Here participants in coherence work need to do things that will help them understand their specific tasks and responsibilities around a set of practices. *For example, nurses recruiting patients into a trial need to have a strong understanding of the work they must do to secure informed consent from patients, and how they will go about this.*
	1.4 Internalization: Finally, sense-making involves people in work that is about understanding the value, benefits and importance of a set of practices. *So, returning to the example of doctors using a videoconferencing system to consult with their patients, it's about the work that they do to attribute worth to a new way of working.*
**2. Cognitive Participation** is the relational work that people do to build and sustain a community of practice around a new technology or complex intervention. Like all NPT constructs, it has four components.	2.1 Initiation: When a set of practices is new or modified, a core problem is whether or not key participants are working to drive them forward. *For example, the work of setting up a clinical service is often delegated to a small group of managers and professionals who are charged with the work of setting up systems, procedures, and protocols and engaging with others to make things happen.*
	2.2 Enrolment: Participants may need to organize or reorganize themselves and others in order to collectively contribute to the work involved in new practices. This is complex work that may involve rethinking individual and group relationships between people and things. *For example, getting nurses to 'buying in' to a falls prevention strategy is vital to its success, but the work of buying in to the strategy is not simply about individual commitment, but is about building communal engagement.*

NPT Core Constructs	
	2.3 Legitimation: An important component of relational work around participation is the work of ensuring that other participants believe it is right for them to be involved, and that they can make a valid contribution to it. *New service interventions often founder because of a lack of investment in ensuring that they fit with the ways that different groups of professionals - and sometimes patients - define their possible contribution to them.*
	2.4 Activation: Once it is underway, participants need to collectively define the actions and procedures needed to sustain a practice and to stay involved. *In fact, staying on the case is vital to sustaining clinical interventions. This is the work of keeping the new practices in view and connecting them with the people who need to be doing them.*
**3. Collective Action** is the operational work that people do to enact a set of practices, whether these represent a new technology or complex healthcare intervention. Like all NPT constructs, it has four components. These were the first NPT constructs to be developed and their names reflect qualities of technologies or complex interventions, rather than the character of the work that these involve.	3.1 Interactional Workability: This refers to the interactional work that people do with each other, with artefacts, and with other elements of a set of practices, when they seek to operationalize them in everyday settings. *For example, a key problem of telemedicine systems has been shown to be their negotiation by doctors and patients as they try to communicate complex clinical information each other over a videoconferencing link.*
	3.2 Relational Integration: This refers to the knowledge work that people do to build accountability and maintain confidence in a set of practices and in each other as they use them. *A telemedicine system that transmitted clinical images of skin lesions ran into trouble when individual doctors began to lose confidence in what these images actually represented, and started to examine patients in parallel to digitized images - thus doubling their workload and putting their clinical department under pressure.*
	3.3 Skill set Workability: This refers to the allocation work that underpins the division of labour that is built up around a set of practices as they are operationalized in the real world. Who gets to do the work is an important element of any set of practices. *For example, a core problem for a research group investigating the effectiveness of a decision aid for medication choice after a a serious illness event was whether the decision aid should be administered by trial managers with no clinical responsibility for the patient, or nurse practitioners actively involved in their care. Allocating the work to the former meant that the decision aid was more easily delivered, but trial managers lacked the clinical expertise of the nurse practitioners which meant that it was hard for them to answer patients' questions.*
	3.4 Contextual Integration: This refers to the resource work - managing a set of practices through the allocation of different kinds of resources and the execution of protocols, policies and procedures. Typically, the implementation of a new set of practices is seen as a management problem, and it's true that the power to allocate resources and define the processes by which new

NPT Core Constructs	
	technologies or complex interventions are executed in practice. The work that is involved in this is about resourcing the ways that others enact a new set of practice.
**4. Reflexive Monitoring** is the appraisal work that people do to assess and understand the ways that a new set of practices affect them and others around them. Like all NPT constructs, it has four components:	4.1 Systematization: participants in any set of practices may seek to determine how effective and useful it is for them and for others, and this involves the work of collecting information in a variety of ways. *The work of systematization may be highly formal - the Randomized Controlled Clinical Trial is a prime example of formal systematization. But it may also be very informal, the collection of anecdotal examples of problems in practice around a set of common themes by an unqualified care assistant is every bit as much an example of the systematization of information.*
	4.2 Communal appraisal: participants work together - sometimes in formal collaboratives, sometimes in informal groups to evaluate the worth of a set of practices. They may use many different means to do this drawing on a variety of experiential and systematized information. *These events happen continuously in almost every setting where people interact around a piece of hardware or new way of organizing work and ask each other 'is it working?' How they put the answers to these questions and negotiate the difficulties that stem from conflicts about what sort of information counts, and how it counts for different groups, are central to the future of any set of practices. Acts of communal appraisal - like data analysis meetings in clinical trials, or quality circles in lean healthcare organizations - are common and may be highly formalized as well as casual and informal.*
	4.3 Individual appraisal: Participants in a new set of practices also work experientially as individuals to appraise its effects on them and the contexts in which they are set. From this work stem actions through which individuals express their personal relationships to new technologies or complex interventions. *For example, a nurse working in a falls prevention program will work to appraise not only the worth of the program, but also its impact on her other tasks. So, a falls program that complicates and adds to an already complicated and demanding workload may well be have a low value attributed to it in practice irrespective of its effects on falls within the hospital.*
	4.4 Reconfiguration: appraisal work by individuals or groups may lead to attempts to redefine procedures or modify practices - and even to change the shape of a new technology itself. *For example, a nurse leading a falls prevention program might look again at the ways in which risk of falling was calculated in practice and the demands that this risk placed on the delivery of nursing care elsewhere on the ward. If the work of calculating risk of falling was disproportionate to the work involved in dealing with other kinds of risks on the ward, then there would be pressure to modify the falls prevention program to make it workable in practice.*

Supplementary Table 3: TDF version 2 domains

[A guide to using the Theoretical Domains Framework of behaviour change to investigate implementation problems | Implementation Science | Full Text (biomedcentral.com)]

TDF Domain (definition and theoretical constructs represented within each domain)
**1. Knowledge** *(An awareness of the existence of something)*<br>1.1. Knowledge *(including knowledge of condition/scientific rationale)*<br>1.2. Procedural knowledge<br>1.3. Knowledge of task environment
**2. Skills** *(An ability or proficiency acquired through practice)*<br>2.1. Skills<br>2.2. Skills development<br>2.3. Competence<br>2.4. Ability<br>2.5. Interpersonal skills<br>2.6. Practice<br>2.7. Skill assessment
**3. Social/professional role and identity** *(A coherent set of behaviours and displayed personal qualities of an individual in a social or work setting)*<br>3.1. Professional identity<br>3.2. Professional role<br>3.3. Social identity<br>3.4. Identity<br>3.5. Professional boundaries<br>3.4. Professional confidence<br>3.5. Group identity<br>3.6. Leadership<br>3.7. Organisational commitment
**4. Beliefs about capabilities** *(Acceptance of the truth, reality or validity about an ability, talent or facility that a person can put to constructive use)*<br>4.1. Self-confidence<br>4.2. Perceived competence

4.3. Self-efficacy<br>4.4. Perceived behavioural control<br>4.5. Beliefs<br>4.6. Self-esteem<br>4.7. Empowerment<br>4.8. Professional confidence
**5. Optimism** *(The confidence that things will happen for the best or that desired goals will be attained)*<br>5.1. Optimism<br>5.2. Pessimism<br>5.3. Unrealistic optimism<br>5.4. Identity
**6. Beliefs about consequences** *(Acceptance of the truth, reality, or validity about outcomes of a behaviour in a given situation)*<br>6.1. Beliefs<br>6.2. Outcome expectancies<br>6.3. Characteristics of outcome expectancies<br>6.4. Anticipated regret<br>6.5. Consequents
**7. Reinforcement** *(Increasing the probability of a response by arranging a dependent relationship, or contingency, between the response and a given stimulus)*<br>7.1. Rewards (proximal/distal, valued/not valued, probable/improbable)<br>7.2. Incentives<br>7.3. Punishment<br>7.4. Consequents<br>7.5. Reinforcement<br>7.6. Contingencies<br>7.7. Sanctions
**8. Intentions** *(A conscious decision to perform a behaviour or a resolve to act in a certain way)*<br>8.1. Stability of intentions<br>8.2. Stages of change model<br>8.3. Transtheoretical model and stages of change
**9. Goals** *(Mental representations of outcomes or end states that an individual wants to achieve)*

9.1. Goals (distal/proximal)<br>9.2. Goal priority<br>9.3. Goal/target setting<br>9.4. Goals (autonomous/controlled)<br>9.5. Action planning<br>9.6. Implementation intention
**10. Memory, attention and decision processes** *(The ability to retain information, focus selectively on aspects of the environment and choose between two or more alternatives)*<br>10.1. Memory<br>10.2. Attention<br>10.3. Attention control<br>10.4. Decision making<br>10.5. Cognitive overload/tiredness
**11. Environmental context and resources** *(Any circumstance of a person's situation or environment that discourages or encourages the development of skills and abilities, independence, social competence and adaptive behaviour)*<br>11.1. Environmental stressors<br>11.2. Resources/material resources<br>11.3. Organisational culture/climate<br>11.4. Salient events/critical incidents<br>11.5. Person × environment interaction<br>11.6. Barriers and facilitators
**12. Social influences** *(Those interpersonal processes that can cause individuals to change their thoughts, feelings, or behaviours)*<br>12.1. Social pressure<br>12.2. Social norms<br>12.3. Group conformity<br>12.4. Social comparisons<br>12.5. Group norms<br>12.6. Social support<br>12.7. Power<br>12.8. Intergroup conflict<br>12.9. Alienation<br>12.10. Group identity

12.11. Modelling
**13. Emotion** *(A complex reaction pattern, involving experiential, behavioural, and physiological elements, by which the individual attempts to deal with a personally significant matter or event)*<br>13.1. Fear<br>13.2. Anxiety<br>13.3. Affect<br>13.4. Stress<br>13.5. Depression<br>13.6. Positive/negative affect<br>13.7. Burn-out
**14. Behavioural regulation** *(Anything aimed at managing or changing objectively observed or measured actions)*<br>14.1. Self-monitoring<br>14.2. Breaking habit<br>14.3. Action planning

TDF: Theoretical Domains Framework. Excerpt from Michie S, Atkins L, West R. The Behaviour Change Wheel: A Guide to Designing Interventions. Great Britain: Silverback Publishing; 2014.

Supplementary Table 4. Coders' characteristics

Coders	Background	Relevant experience	Years of research and/or clinical experience
ARN	Public health and Environment Researcher	Lead author of the initial systematic review, mixed methods researcher. Methodological and applied expertise in public health and environmental science research.	25 years
HA	Primary care Researcher	Co-author of initial systematic review, expertise in primary care research, experienced in use of NPT, mixed methods researcher.	20 years
FD	Management and Organisational Studies Researcher	Qualitative researcher focused on organisational responses to climate change	19 years
AE	Researcher	Co-author of initial systematic review. Qualitative health researcher	14 years
OG	Research Associate	Co-author of initial systematic review. Qualitative health researcher	1.5 years
MG	Patient and Public Contributor	PPI Co-Investigator on the GPNET-0 study. Co-author of initial systematic review. Supports research projects from planning stages through to dissemination. Co-chair of patient-focussed EDI group in local NHS.	4.5 years
FK	Research Fellow	Co-author of initial systematic review, qualitative organisation studies researcher.	11 years
RS	Academic GP	Co-author of initial systematic review, clinician, mixed methods researcher, experience with complex intervention design/delivery.	19 years
HT	Academic GP	Co-author of initial systematic review, clinician, mixed methods researcher, member of Greener Practice.	20 years
JD	Academic GP	Co-author of initial systematic review, clinician, mixed methods researcher, experience with complex intervention design/delivery.	42 years
